# Qizhijiangtang capsule for the treatment of diabetic kidney disease: A protocol for systematic review and meta-analysis

**DOI:** 10.1097/MD.0000000000021923

**Published:** 2020-08-21

**Authors:** Yumeng Tan, Jun Hu, Yueying Zhang, Qian Wu, Qing Ni

**Affiliations:** a Department of Endocrinology; b Department of Cardiovascular, Guang’anmen Hospital, China Academy of Chinese Medical Sciences; c Beijing University of Chinese Medicine, Beijing, China.

**Keywords:** diabetic kidney disease, meta-analysis, protocol, qizhijiangtang capsule

## Abstract

**Backgrounds:**

Diabetic kidney disease (DKD) is 1 of the common microvascular complications of diabetes, and the therapeutic effect of modern medicine on DKD is limited. At present, patented Chinese medicine Qizhijiangtang (QZJT) capsule has been widely used in the treatment of DKD. We aim to systematically assess the efficacy and safety of QZJT capsule for the treatment of diabetic kidney disease (DKD).

**Methods:**

Randomized controlled trials of QZJT capsule for DKD treatment will be searched until July 1, 2020, in 7 electronic databases: PubMed, Embase, Cochrane Library, CNKI, Wanfang, VIP, and Chinese Biomedical Literature. Furthermore, additional relevant publications will be manually searched according to reference lists from the resulting publications. The Cochrane risk test from the Cochrane Handbook will be used as a bias tool to evaluate the methodological quality. The clinical efficacy will be the primary outcome, which is based on the changes in symptoms and levels of proteinuria. Review Manager 5.3 will be used to analyze the results.

**Results and conclusions:**

Our meta-analysis will provide evidence to the clinical application of QZJT capsule in the treatment of DKD from the 4 aspects including the clinical efficacy, changes in proteinuria, the renal function and level of blood glucose. Meanwhile, the results can also reflect the role of traditional Chinese medicine in the treatment of DKD.

**PROSPERO registration number:**

CRD42020153949.

## 1 Introduction

Diabetic kidney disease is 1 of the common microvascular complications of diabetes mellitus (DM), as well as a key cause of end-stage renal disease.^[1,2]^ Researches abroad show that 20%∼40% of diabetics with diabetic kidney disease (DKD), and the prevalence rate of DKD in type2 DM in China is 10% to 40%.^[3–6]^ Once DKD progresses into the clinical stage, the development of kidney damage in DKD patients will be rapid. At present, there is no effective means to intervene the kidney injury, so the majority of DKD patients will become end-stage renal disease in a relatively short time.^[7]^ Modern medicine mainly treats DKD through the following measures: glycemic control, blood lipid regulation, proteinuria lowering and hemodialysis, which play an important role in delaying DKD progression, but the efficacy is still very limited.^[8]^

Compared with modern medicine, traditional Chinese medicine (TCM) treats DKD of little side effects, significantly improving patients’ clinical symptoms and quality of life. TCM has a definite effect on delaying the progress of DKD and has great clinical application prospect. Qizhijiangtang (QZJT) capsule is a compound Chinese patent medicine composed of radix Astragali, hirudo, radix rehmanniae and rhizoma polygonatum. Studies have shown that QZJT capsule can not only improve the insulin resistance and regulate blood glucose in diabetic rats,^[9]^ but also alleviate DKD rats’ pathological damage of kidney tissue and blood vessels.^[10]^ Now, QZJT capsule has been widely used in the treatment of type2 DM and DKD.

Although several clinical studies have confirmed the efficacy of QZJT capsule for DKD,^[11–13]^ most of the studies include small cohorts and different treatment schemes. Therefore, it is essential to conduct systematic review of these studies. The aim of our meta-analysis is to assess the efficacy and safety of QZJT capsule for the treatment of DKD, providing evidence for clinical practice.

## 2 Methods

We have registered this protocol on PROSPERO as CRD42020153949, and will perform it according to the Preferred Reporting Items for Systematic Reviews and Meta-Analysis Protocol statement guidelines.

### 2.1 Data sources and search strategy

randomized controlled trial (RCTs) of QZJT capsule for DKD therapy will be searched until July 1, 2020, in 7 electronic databases: PubMed, Embase, Cochrane Library, CNKI, Wanfang, VIP, and Chinese Biomedical Literature. Furthermore, additional relevant publications will be manually searched according to reference lists from the resulting publications. The search terms are as follows: diabetic kidney disease or diabetic nephropathy, QizhiJiangtang or QizhiJiangtang capsule, RCT or controlled clinical trial or random or randomly. We will apply different search strategies to Chinese and foreign language databases, without restriction on language or publications.

### 2.2 Study selection and inclusion criteria

#### 2.2.1 Types of studies

RCTs of humans using QZJT capsule for the treatment of DKD will be included. All included studies should clearly report the random methods, diagnostic criteria and efficacy evaluation criteria that they adopted. Studies with erroneous or incomplete data will be excluded.

#### 2.2.2 Types of patients

All included patients have been definitely diagnosed with DKD. There are no limitations in the type of DM, stage of the DKD, age, gender, or race.

#### 2.2.3 Types of interventions

The experimental group is treated with QZJT capsule without limitation in dosage, or combined with western medicine. There is no restriction on interventions in control groups, whether placebo, no therapy, or western medicine. But, both groups don’t use other TCM treatment, such as TCM decoction, herbal extracts, acupuncture or other Chinese patent medicine.

#### 2.2.4 Types of outcomes

The clinical efficacy will be the primary outcomes, which is based on the changes in symptoms and levels of proteinuria. The clinical efficacy was categorized as significantly effective cases (urinary albumin excretion rate [UAER] returned to normal levels or decreased by more than 50%, with an obvious improvement in symptoms), effective cases (UAER decreased by less than 50%, improvement in symptoms), or ineffective cases (no improvement in either UAER and symptoms).^[14]^ The secondary outcomes will include the proteinuria indicators UAER, and urinary albumin creatinine ratio; the renal function indicators estimated glomerular filtration rate, blood urea nitrogen, and serum creatinine; and hemoglobin A1c.

### 2.3 Data collection and extraction

**Figure 1:**
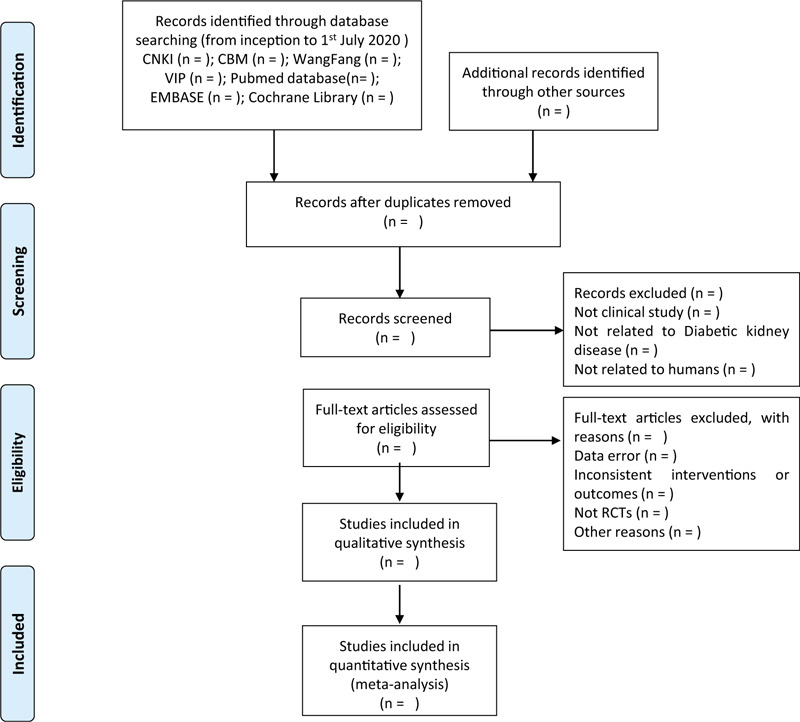
Flow chart of the literature screen.

All eligible studies and useful data will be collected and extracted by 2 researchers independently. The flow chart of the literature screen will be shown in Figure 1. First, they will screen the titles and abstracts of all records obtained from electronic databases and other source by Endnote software. Then, they will conduct further assessment of remaining records through reading full-text for eligibility. Finally, the data including study ID, baseline indicators, disease data, interventions, and outcomes (eg, sample size, age, gender, type of DM, stage of DKD, interventional measures, treatment duration, reporting of adverse events, and outcome measures) will be extracted by them. Any discrepancies in the process will be resolved by discussion with other authors.

### 2.4 Quality assessment

Two researchers will assess the risk of bias independently according to the Cochrane Handbook for the methodological quality of the included studies. They will use the Review Manager 5.3 (Cochrane Collaboration, Oxford, UK) to evaluate the following 6 items: random sequence generation (selection bias), allocation concealment (selection bias), blinding of participants and personnel (performance bias), blinding of outcome assessment (detection bias), incomplete outcome data (attrition bias), selective reporting (reporting bias), and other sources of bias such as baseline comparability of subjects and sample size.^[15]^ Each item will be categorized into 3 levels: high risk, low risk or unclear. Any disagreements will be resolved by a third party (Qing Ni).

### 2.5 Data analysis

We will apply Review Manager 5.3 software for statistical analysis. Dichotomous data will be expressed as relative risk, and continuous data will be presented as the mean difference. Both kinds of data will be included a 95% confidence interval. The statistical heterogeneity assessments will be conducted using a Chi2 test. We will use the fixed-effects model when the heterogeneity was significant (*P* ≥ .10, *I*^2^ ≤ 50%), otherwise a randomized effects model will be used (ie, when *P* < .10, *I*^2^ > 50%). Then, the possible sources of heterogeneity will be explored by sensitivity analysis and subgroup analysis. We plan to do the following subgroup analyses if possible: comparison between QZJT capsule only and QZJT capsule plus western medicine; comparison between different treatment durations; comparison between different stages of DKD. Publication bias will be tested using funnel plots.

## 3 Discussion

Diabetic kidney disease, as 1 of the most important complication of diabetes, can significantly increase the risk of cardiovascular disease and all-cause death in diabetic patients,^[16]^ while effective treatment can improve the survival rate and life quality of DM patients. The occurrence and development of DKD is the result of multi-factor interactions, and the comprehensive treatment measures involve targeting hypoglycemia and hypotension as well as the reduction of proteinuria. Renin-angiotensin-aldosterone system inhibitors and sodium-glucose co-transporter 2 inhibitors are currently the few drugs with evidence of kidney protection, but the incidence of renal endpoint events is still high.^[17]^ Thus, it is necessary to explore additional intervention methods to counter DKD.

Recently, there are more and more clinical studies on QZJT capsule for DKD. The results of these studies suggest that QZJT capsule may be a potentially effective therapy for DKD,^[11–13]^ but no definite conclusion has been reached yet. However, the Chinese medicines that make up QZJT Capsule have been proved to protect the kidney by pharmacological studies. Among them, Astragalus iv and Astragalus polysaccharides in Astragalus can reduce the damage of renal tubules and podocytes, inhibit renal interstitial fibrosis.^[18–20]^ Hirudus can improve renal microcirculation, reduce ischemia-hypoxia damage in DKD rats.^[21]^ Catalpa in radix rehmanniae can inhibit the expression of Grb10 and improve the renal function injury of DKD rats.^[22]^ Therefore, it's essential to do the meta-analysis of currently published studies to assess the effectiveness of QZJT capsule for treating DKD.

However, our meta-analysis may have some limitations. If the included studies have problems such as poor methodological quality, small sample size, lacking of follow-up and adverse events reports, the conclusions of the review will be uncertain, especially regarding the evaluation of the long-term efficacy and safety of QZJT capsule for DKD. In that case, we will need more high-quality, large-scale, and multicenter RCTs for further verification.

In a word, this meta-analysis will provide evidence to the clinical application of QZJT capsule in the treatment of DKD, and also reflect the role of TCM in the treatment of DKD.

## Author contributions

**Data curation:** Qian Wu, Yueying Zhang,

**Methodology:** Qing Ni, Yumeng Tan, Jun Hu.

**Project administration:** Yumeng Tan.

**Supervision:** Qing Ni.

**Writing – original draft:** Yumeng Tan, Jun Hu.

**Writing – review & editing:** Yumeng Tan, Jun Hu.
